# Risk factors for adverse events occurring after recovery from stereotactic brain biopsy in dogs with primary intracranial neoplasia

**DOI:** 10.1111/jvim.15885

**Published:** 2020-09-14

**Authors:** Richard L. Shinn, Yukitaka Kani, Fang‐Chi Hsu, John H. Rossmeisl

**Affiliations:** ^1^ Veterinary and Comparative Neuro‐oncology Laboratory, Department of Small Animal Clinical Sciences Virginia‐Maryland College of Veterinary Medicine, Virginia Tech Blacksburg Virginia USA; ^2^ Departments of Biostatistical Sciences Wake Forest University Winston‐Salem North Carolina USA; ^3^ Comprehensive Cancer Center and Brain Tumor Center of Excellence, School of Medicine Wake Forest University Winston‐Salem North Carolina USA

**Keywords:** brain tumor, canine, diagnostic methods, neurosurgery, risk factors

## Abstract

**Background:**

Stereotactic brain biopsy (SBB) allows for histopathologic diagnosis of brain tumors. Adverse events (AE) occur in 5 to 29% of dogs after SBB, but risk factors associated with developing AE are poorly described.

**Objective:**

Identify clinicopathologic, diagnostic imaging, or procedural variables that are associated with AE in dogs after SBB.

**Animals:**

Twenty‐nine dogs with brain tumors.

**Methods:**

Retrospective, case‐control study. Dogs had laboratory investigations performed before SBB, as well as clinical examinations and diagnostic imaging of the brain before and after SBB. Cases experienced AE after SBB including transient exacerbation of preexisting neurologic deficits, transient new deficits, or permanent neurologic deficits. Controls had SBB performed without AE. Fisher's exact and Student's *t* tests were used to examine associations between the postulated risk factors and AE.

**Results:**

Adverse events occurred in 8/29 (27%) dogs, and 7/8 AE (88%) were transient. Cases were significantly more likely to have T2W‐heterogenous tumors (88 versus 38%; *P* = .04) and lower platelet counts (194.75 ± 108.32 versus 284.29 ± 68.54 ×10^3^/mm^3^, *P* = .006). Dogs with gradient echo signal voids present on baseline imaging were significantly more likely to have hemorrhage present after biopsy, and 7/8 (88%) of cases had hemorrhage on imaging after SBB.

**Conclusion and Clinical Importance:**

Twenty‐seven percent of dogs undergoing SBB experience AE, with the majority of AE resolving with 1 week. Platelet counts should be ≥185 000/mm^3^ to minimize risk of SBB‐associated AE. Observation of intracranial hemorrhage after biopsy can have important clinical implications, as this was observed in 88% of dogs with AE.

**Abbreviations**
AE: adverse events
AUC: area under the curve
CT: computed tomography
KPS: Karnofsky Performance Score
MRI: magnetic resonance imaging
SBB: stereotactic brain biopsy

## 1 INTRODUCTION

Magnetic resonance imaging (MRI) has transformed the way companion animals with intracranial lesions are managed but still has considerable limitations in the definitive diagnosis of brain diseases.^1^, ^2^ Histopathologic examination is often needed for definitive diagnosis of intracranial lesions. In dogs with brain lesions that cannot be safely or practically sampled using excisional biopsy, minimally invasive techniques, such as stereotactic brain biopsy (SBB), can be used to obtain brain tissue for definitive diagnosis.^3^, ^4^ Although clinical use of SBB for antemortem histologic diagnosis of intracranial lesions is becoming more widespread in veterinary medicine, there is still limited knowledge about the types of adverse events (AE) attributed to SBB, and risk factors for their development.^3^, ^5^ In humans, 2.2 to 8.8% of patients will experience neurologic deterioration after SBB, 0.7% will have permanent neurologic deficits, and the risk of death is between 0 and 2.8%.^6^, ^7^, ^8^, ^9^, ^10^ In dogs, the frequencies of AE after SBB range from 12 to 29%, with the majority of AE described involving transient neurological deterioration that resolves within 2 weeks, and 5 to 9% of dogs dying.^11^, ^12^, ^13^, ^14^, ^15^

Neurologic deterioration after SBB is well documented in people. It is frequently observed within the first 2 hours after biopsy, and is often associated with postoperative hemorrhage seen on computed tomography (CT).^10^ Humans with a platelet count <150 000/mm^3^ are at increased risk for biopsy‐associated hemorrhage and the development of AE.^7^, ^8^, ^16^ Similar predictors of SBB risk have not been evaluated in dogs.

The goals of this study are to identify clinical, pathologic, diagnostic imaging, or biopsy technical variables that are associated with the development of AE in dogs subjected to SBB. We hypothesized that the presence of new intralesional hemorrhage on postbiopsy imaging studies is positively associated with the development of AE. Other hypotheses evaluated included: the presence of preexisting intralesional hemorrhage is associated with greater risk of developing postbiopsy hemorrhage; a longer needle path length through brain tissue will be more likely to be associated with AE; dogs with strongly contrast enhancing tumors will be more likely to develop AE; dogs with more severe neurological dysfunction (lower Karnofsky Performance Scores [KPS]) will be more likely to experience AE, and the platelet counts of dogs that experienced AE will be lower than those that did not.

## 2 MATERIALS AND METHODS

### 2.1 Study design

Retrospective, case‐control study.

### 2.2 Inclusion criteria and risk factors examined

The medical subject heading terms biopsy, brain neoplasm, craniotomy, dogs, intracranial hemorrhages, intraoperative complications, pneumocephalus, and stereotaxic techniques were used to retrospectively search a medical records database over the period of 2009 to 2020 to identify dogs with brain tumors that underwent SBB. Cases were those dogs with brain tumors that experienced an AE attributed to the SBB procedure, and controls those dogs that had SBB performed without complication.

To be included in the study, each dog was required to have a diagnostic MRI examination of the brain performed within 2 weeks before the SBB available for review; a postbiopsy CT or MRI of the of the brain obtained within 72 hours of SBB and before the performance of any additional intracranial neurosurgical interventions; a complete blood count and serum biochemical profile performed <24 hours before the SBB; and to have been examined by a board‐certified neurologist before the SBB and daily until hospital discharge after SBB. A single neurosurgeon performed all SBB in anesthetized dogs using a previously described technique and custom small animal headframe.^13^ A single needle trajectory was used to obtain SBB specimens from all dogs. After SBB, dogs were monitored for a minimum of 48 hours for evaluation of AE. For the purposes of this study, AE were categorized as: (1) transient exacerbation of preexisting neurological deficit; (2) transient new neurological deficit; or (3) permanent neurological deficit. Transient neurological deficits were defined as those that resolved spontaneously or with corticosteroid treatment within 7 days, and permanent neurological deficits as those that persisted for >7 days after SBB.

Preoperative MRI results were obtained from several referring veterinary practices. As such, images were generated using low‐ and high‐field magnets (0.2‐3.0T), and sequences and image acquisition parameters were not standardized. All preoperative MRI datasets contained precontrast T2W and T1W images in at least 2 planes, at least a single planar T2W fluid attenuated inversion recovery (FLAIR) sequence, and postcontrast T1W images in at least 2 planes. All dogs in which postbiopsy MRI were performed had standardized sequences obtained on a 1.5T magnet (Intera; Toshiba, Japan): pre‐ and postcontrast (0.1 mmol/kg gadopentetate dimeglumine IV; Magnevist, Bayer HealthCare) 3‐dimensional T1W (3DT1W), T2W (sagittal, dorsal, and transverse), and transverse diffusion weighted, FLAIR, and T2 gradient echo.^17^ Additional sequences were obtained at the discretion of the attending radiologist. Stereotactic planning and postbiopsy CT scans were performed with a 16‐slice helical scanner (Aquilion; Toshiba, Japan). All dogs had transverse, 1‐mm contiguous axial CT slices obtained from the nares through the level of C2 using 120 kV and 300 mA settings with a field of view that included the entire stereotactic headframe apparatus. Postcontrast CT images were obtained after IV administration of 2 mL/kg of non‐ionic, iodinated contrast medium (Iohexol, 300 mg I/mL, Nycomed, Princeton, New Jersey) delivered over 20 seconds using a power injector.

Data extracted from medical records included the breed, age, weight, sex, body condition score (BCS), type and duration of clinical signs before presentation, duration of corticosteroid treatment before presentation, type of anticonvulsant treatment the dog was receiving, neurolocalization, and KPS (Supplemental File 1) before SBB.^18^, ^19^, ^20^, ^21^ Clinicopathologic variables that were recorded included the tumor type, grade, and location within the brain, red blood cell (RBC) count, platelet count, total white blood cell (WBC) count, creatine kinase (CK), albumin, globulin, alanine transaminase (ALT), and alkaline phosphatase (ALP).^14^

Pre‐ and postbiopsy diagnostic imaging studies were independently reviewed by 2 investigators blinded to the biopsy and clinical outcome results, and the following data recorded as previously described: the total T2W tumor volume, the total T2W brain volume, the total tumor:brain volume, tumor shape (ill‐defined, ovoid, or spherical) and margination (smooth or irregular), growth pattern (focal or diffuse), intrinsic T1W and T2W tumor signal characteristics (hypointense, isointense, hyperintense, or heterogeneous), and intrinsic CT attenuating appearance of tumor parenchyma (hypoattenuating, isoattenuating, hyperattenuating, or heterogeneous).^1^, ^13^, ^20^, ^22^, ^23^, ^24^ Tumors were considered homogeneous if ≥90% of the tumor mass displayed uniform signal or attenuation features; tumors not meeting this criteria were classified as heterogeneous.^22^ The following imaging features were scored as present or absent: intralesional T2*GRE signal voids, intratumoral fluid, peritumoral edema, midline shift >3 mm, ventricular distortion, subfalcine, transtentorial, and foramen magnum herniations, and the presence of new intracranial hemorrhage or air after the SBB. Hemorrhagic areas were defined by hypointensity on T2* gradient echo sequences, or hyperattenuating regions on precontrast CT scans.^20^, ^22^, ^24^ The pattern (heterogeneous, homogeneous, or ring) and severity (none, mild, moderate, severe) of contrast enhancement was also scored on postcontrast CT and MRI studies.^1^, ^13^, ^22^, ^23^, ^24^ In instances in which there was discordance between the 2 observers regarding imaging findings, a third investigator reviewed the data and consensus was used to assign the final value. Biopsy technical variables that were recorded included the number of biopsies obtained and the maximum biopsy needle path length, as measured from the top of the skull at the margin of the craniotomy portal of needle entry to the tip of the biopsy needle when inserted into the deepest aspect of the target.^1^, ^13^

### 2.3 Statistical analyses

Means, standard deviations, medians, and ranges were calculated for continuous characteristics. Counts and proportions were calculated for discrete characteristics. Student's *t* tests were used to compare continuous demographic, tumor, and laboratory variables between case and control dogs, and Fisher exact tests used to compare nominal diagnostic imaging variables between the 2 groups. Area under the curve (AUC) statistics of the receiver operating characteristic (ROC) curve were used to assess the prediction performance of the platelet count to discriminate case and controls dogs. The AUC and its confidence interval were estimated, and the sensitivity and specificity were calculated. Statistical analyses were completed using SAS software version 9.4 (SAS Institute, Cary, North Carolina). *P*‐values <.05 were considered significant.

## 3 RESULTS

Over the study period, 59 SBB were performed in dogs with brain tumors, and 96.6% (57/59) of dogs recovered and survived to hospital discharge. Between the 2 deaths, 1 (1.7%) occurred in a case in which craniotomy and transfusion were required control intracranial hemorrhage that was initiated by SBB of a high‐grade oligodendroglioma, and 1 (1.7%) occurred in a dog that developed aspiration pneumonia after SBB and contemporaneous glioma treatment.^13^ Records of 30 dogs were excluded because of performance of a therapeutic neurosurgical intervention immediately after SBB that precluded evaluation of biopsy‐related AE (n = 21), performance of the baseline MRI study >4 weeks before SBB (n = 4), absence of brain imaging performed within 72 hours of SBB (n = 4), and death immediately after SBB and exploratory craniotomy before anesthetic recovery (n = 1).

Twenty‐nine dogs were included in the study (Tables 1 and 2), which included 28 dogs with gliomas and 1 dog with a meningioma. All dogs included in the study had structural epilepsy, and 20/29 (69%) had additional interictal neurological deficits referable to the location of their brain tumor. In total, 8/29 (27%) dogs developed AE after SBB (cases) and 21/29 (73%) did not (controls). Adverse events were characterized by transient exacerbation of preexisting neurological deficits in 6/8 (75%) cases (6/29 [20.7%] of all dogs), and transient new neurological and permanent neurological deficits in 1/8 (12.5%) cases each (1/29 [3%] of all dogs experienced either transient new or permanent neurologic deficits). Both the transient new and permanent AE were characterized by the development of thalamocortical visual deficits referable to the brain ipsilateral to the side on which SBB was performed. The permanent AE was present for at least 62 days, which was duration of follow‐up for the case in which it occurred. None of the neurological AE observed required prolongation of hospitalization, delay in the time to tumor treatment, or specific or invasive interventions to treat. Breeds included Boxer (n = 8), Boston Terrier (n = 5), Staffordshire terrier (n = 4), English bulldog (n = 2), mixed breed (n = 2), and 1 of each of the following: Cane Corso, Catahoula Leopard, French Bulldog, Golden Retriever, Labrador Retriever, Miniature Schnauzer, Portuguese Waterdog, and Shar‐Pei. Fourteen dogs were altered males, 11 dogs were altered females, 1 dog was an intact male, and 3 dogs were intact females. The age, weight, and KPS scores were 8.2 ± 1.8 years (mean, SD), 25 ± 11.1 kg, and 80.5 ± 9.5, respectively.

**TABLE 1:** Demographic and clinicopathological variables in cases (dogs that experienced adverse events) and controls (dogs that did not experience adverse events) with primary brain tumors subjected to stereotactic brain biopsy

	Cases (n = 8)	Controls (n = 21)	*P* value
Subject variables^a^			
Age (years)	8.5 (1.5)	8.0 (1.8)	.45
Weight (kg)	21.8 (12.8)	24.8 (10.6)	.51
Baseline Karnofsky Performance Score	77 (10.4)	81.7 (9.1)	.34
Tumor type and grade (%; proportion)			
Low‐grade astrocytoma	12 (1/8)	10 (2/21)	.82
High‐grade astrocytoma	25 (2/8)	29 (6/21)	.74
Low‐grade oligodendroglioma	0	19 (4/21)	.25
High‐grade oligodendroglioma	63 (5/8)	33 (7/21)	.16
Low‐grade unclassified glioma	0	5 (1/21)	.72
Meningioma	0	5 (1/21)	.72
Tumor volumes^a^			
Tumor volume (cm^3^)	4.39 (1.47)	3.94 (3.30)	.28
Tumor:brain volume ratio	.059 (.014)	.044 (.032)	.09
Biopsy technical variables			
Biopsy needle path length (mm)^a^	36 (15.7)	29.9 (13.3)	.30
Number of biopsies (median, range)^b^	2 (1–3)	2 (1‐5)	.19
Laboratory variables^a^			
Albumin (g/dL)	3.49 (.29)	3.21 (.36)	.09
Globulin (g/dL)	3.33 (.36)	3.15 (.43)	.44
ALT (U/L)	21 (77)	214 (337)	.92
ALP (U/L)	1052 (1400)	912 (1731)	.55
Creatine kinase (U/L)	122.3 (29.7)	140.4 (47.4)	.62
RBC (×10^6^ cells/mm^3^)	6.726 (.99)	6.554 (.71)	.77
WBC (×10^3^ cells/mm^3^)	12.88 (3.64)	10.82 (3.77)	.16
Platelets (×10^3^ cells/mm^3^)	194.75(108.32)	284.29(68.54)	.006*

^a^ Data presented as mean (SD).

^b^ Data presented as median (range).

* Statistically significant, *P* < .05.

**TABLE 2:** Diagnostic imaging findings in cases (dogs that experienced adverse events) and controls (dogs that did not experience adverse events) with primary brain tumors before (baseline) and after stereotactic brain biopsy

	Cases (n = 8)	Controls (n = 21)	*P* value
Baseline brain magnetic resonance imaging (MRI) characteristics (%; proportion)			
Tumor contrast enhancement			
Contrast pattern (all patterns)	Various	Various	.67
No contrast enhancement	12 (1/8)	19 (4/21)	.82
Heterogenous contrast enhancement	0	14 (3/21)	.54
Complete or partial ring enhancement	88 (7/8)	66 (14/21)	.26
Tumor signal characteristics			
T2W‐heterogeneity	88 (7/8)	38 (8/21)	.04*
T1W‐hypoinetense	50 (4/8)	71 (15/21)	.39
T1W‐heterogeneity	38 (3/8)	29 (6/21)	.67
Gradient echo signal voids	50 (4/8)	38 (8/21)	.68
Other tumor characteristics			
Peri‐tumoral edema	100 (8/8)	81 (17/21)	.55
Frontal location	38 (3/8)	29 (6/21)	.67
Parietal location	25 (2/8)	24 (5/21)	1.00
Temporal location	25 (2/8)	14 (3/21)	.60
Piriform location	12 (1/8)	33 (7/21)	.38
Spherical or ovoid shape	38 (3/8)	57 (12/21)	.43
Intratumoral fluid	12 (1/8)	9 (2/21)	1.00
Features of mass effect			
Distortion of lateral ventricle	100 (8/8)	61 (13/21)	.066
Abuts ventricle	75 (6/8)	71 (15/21)	1.00
Falcine herniation or midline shift > 3 mm	25 (2/8)	29 (6/21)	1.00
Transtentorial herniation	37.5 (3/8)	38 (8/21)	1.00
Foramen magnum herniation	25 (2/8)	24 (5/21)	1.00
Postbiopsy imaging findings (%; proportion)			
Intracranial hemorrhage	88 (7/8)	52 (11/21)	.08
Pneumocephalus	12 (1/8)	19 (4/21)	.67

* Statistically significant, *P* < .05.

**FIGURE 1:**
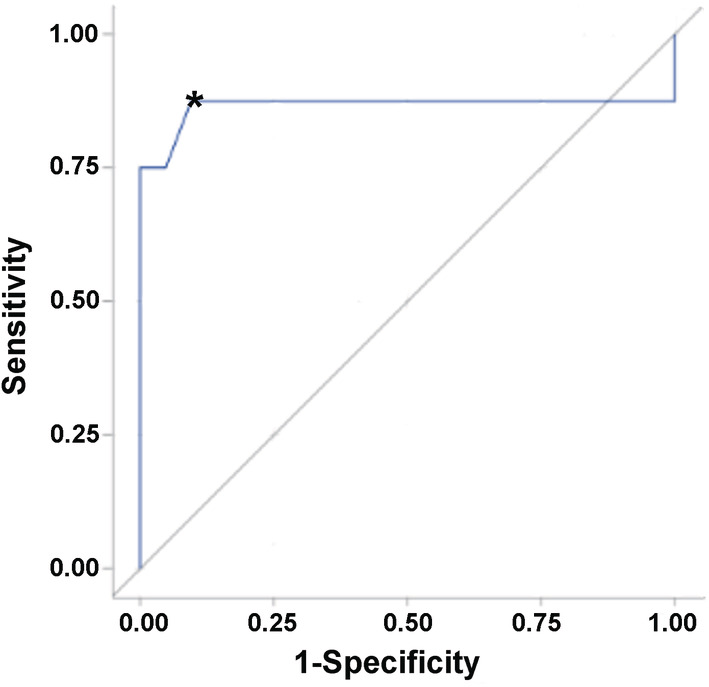
Receiver operating characteristic (ROC) curve for platelet count in dogs experiencing adverse events (AE) after stereotactic brain biopsy versus those that did not develop AE. The black diagonal line represents a completely uninformative test, wherein the area under the curve (AUC) is 50%. The area under the ROC curve represented by the blue line is 0.866 (95% confidence interval, 0.62‐1.0). A platelet count of 185 000/mm^3^ (*) optimally discriminated dogs that developed AE after stereotactic brain biopsy (SBB) from those that did not, with a corresponding sensitivity and specificity of 87.5 and 78%, respectively

There were no features of the dog's signalment, tumor volume, tumor type and grade, or biopsy technical variables that were significantly associated with the development of AE (Table 1). The platelet count was the only laboratory variable associated with AE, and was significantly lower (*P* = .006; Table 1) in cases. An ROC curve was constructed for the platelet count (Figure 1). The ROC AUC was 0.866 (95% confidence interval, 0.623‐1.0). An optimal platelet count of 185 000/mm^3^ (reference range, 131 000‐484 000/mm^3^) was identified from the ROC curve, with a corresponding sensitivity and specificity of 87.5 and 78%, respectively.

**FIGURE 2:**
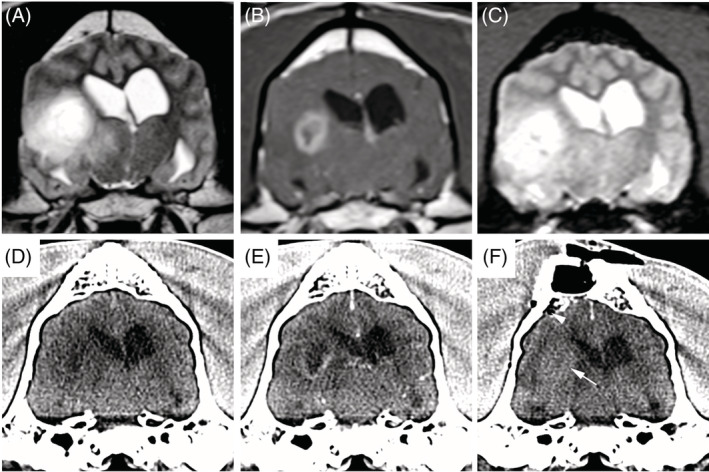
Pneumocephalus and iatrogenic intratumoral hemorrhage after stereotactic brain biopsy in a case dog that developed adverse events. Transverse prebiopsy MRI (A, precontrast T2W; B, postcontrast T1W; and C, T2*gradient echo) and transverse stereotactic planning CT (D, precontrast; E, postcontrast) of a high‐grade astrocytoma in the temporal lobe demonstrating T2W heterogeneous signal (A) and marked ring enhancement (B). There is no evidence of intracranial hemorrhage on the prebiopsy images (A‐E), while hyperattenuating intratumoral hemorrhage (F; arrow) and a small focus of subarachnoid gas (arrowhead) are visible on the postbiopsy CT scan

**FIGURE 3:**
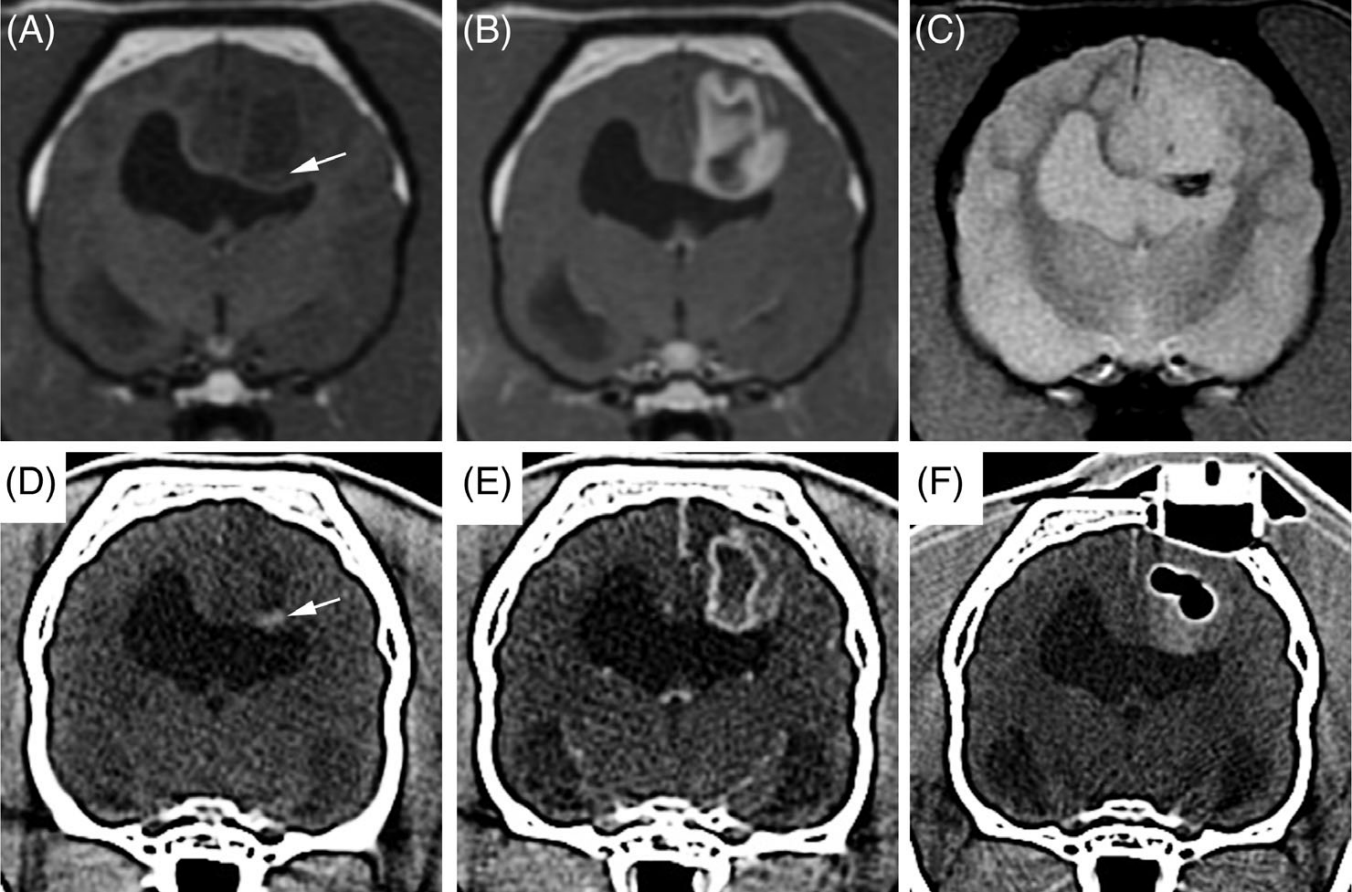
Intraparenchymal pneumocephalus and exacerbation of preexisting intratumoral hemorrhage after stereotactic brain biopsy in a control dog that did not experience adverse events. Transverse prebiopsy MRI (A, precontrast T1W; B, postcontrast T1W; and C, T2* gradient echo) and transverse stereotactic planning CT (D, precontrast; E, postcontrast) of a markedly ring‐enhancing high‐grade astrocytoma in the parietal lobe. Spontaneous intratumoral hemorrhage appears a focus of T1W hyperintensity (A, arrow), T2* gradient echo single void (C), and hyperattenuating lesion on CT (D, arrow) in the ventral aspect of the tumor on the prebiopsy images. On the postbiopsy CT scan (F), a multilobular pocket of gas is surrounded by hyperattenuating intratumoral hemorrhage

All dogs had a CT scan of the brain after SBB, and 8/29 also had an MRI performed. The median time to the performance of postbiopsy CT was 0.6 hours (range, 0.3‐2.8 hours), and the median time to postbiopsy MRI was 59.2 hours days (range, 44.3‐87.5 hours). Abnormalities frequently observed on postbiopsy imaging studies included intracranial hemorrhage (18/29 dogs) and air (5/29 dogs; Figures 2 and 3). All imaging abnormalities that were identified in the 8 dogs that underwent postbiopsy MRI were also present on the postbiopsy CT, and no new or progressive intracranial hemorrhage were noted in dogs that had postbiopsy MRI performed. Among diagnostic imaging variables examined (Table 2), cases were significantly more likely to have a T2W‐heterogeneous tumor (*P* = .04) compared to controls, and dogs with gradient echo signal voids present on prebiopsy MRI were significantly more likely to have hemorrhage present on postbiopsy imaging studies (*P* = .006). No other significant differences in diagnostic imaging features were observed between case and controls (Table 2).

## 4 DISCUSSION

We observed AE characterized by the exacerbation of preexisting or the development of new neurological deficits in 27% of dogs in which SBB was performed, and 88% of these AE were transient. Variables identified as significant risk factors for SBB associated AE included the platelet count and heterogeneous T2W tumor signal.

As biopsy induced hemorrhage is directly related to morbidity and death in humans undergoing SBB, it is recommended that the platelet count be >100 000/mm^3^ before SBB, but the risk of hemorrhage steadily increases as the platelet count falls below 150 000/mm^3^.^7^, ^8^, ^9^ Currently, there are no evidenced based guidelines for what the platelet count should be in dogs if SBB is to be performed, although the recommendation for surgery in general is that the platelet count should be ≥50 000/mm^3^.^25^ In this study, 7/29 (24%) of dogs had platelet counts <185 000/mm^3^, and all 7 of these cases developed AE and had evidence of intralesional hemorrhage present on postbiopsy diagnostic imaging. Although the presence of intracranial hemorrhage after biopsy was not significantly associated with AE in our study, our findings suggest there could be a correlation between platelet counts and the development of clinically relevant intracranial hemorrhage, and this relationship should be investigated in a larger population of dogs subjected to SBB. Our results indicate that intracranial hemorrhage after SBB is clinically silent in many dogs, but also stress that platelet counts required to prevent clinically important intracranial hemorrhages may be greater than those required to mitigate bleeding risk in other soft‐tissue surgical procedures. This may be because of the highly vascular nature of the normal brain or many glial tumors.^26^, ^27^

Currently, there are no recommendations for how long dogs should be monitored for delayed onset AE after SBB. In people, some reports suggest that in the absence of hemorrhage on postbiopsy diagnostic imaging studies, discharge within 8 hours of SBB is safe.^9^ Other studies report that delayed or initially silent hemorrhage after SBB can take up to 48 hours to clinically manifest.^8^, ^16^ At our practice, dogs are routinely kept for a minimum of 48 hours after SBB. Within this time period, no dog developed clinical signs consistent with delayed hemorrhage or other AE. Based on our results, performing CT imaging of the brain immediately after SBB is a reasonable approach to screen for dogs who have postbiopsy hemorrhage and could be at risk for associated AE.

Dogs with T2W‐heterogenous tumors were more likely to have postbiopsy AE. Although the reason for this is not known, it is speculated to be related to higher grade tumors. T2W‐heterogenicity has been associated with higher grade glial tumors in dogs but this feature does not reliably discriminate tumor grades, and we did not identify significant associations between AE and tumor grade or type.^22^, ^24^ Ring‐enhancement is also more common in high‐grade gliomas, but was also not a significant risk factor for AE in our study.^22^ Gliomas can often be heterogenous in nature and small biopsy samples may not reflect geographic tumor histology.^1^, ^27^ Therefore, it is possible that some of the tumors were not correctly graded, but more likely reflects overlapping MRI characteristics between tumor grades.^20^, ^21^, ^22^

Dogs were also more likely to have hemorrhage present on postbiopsy diagnostic imaging if gradient echo signal voids were present on MRI exams obtained before biopsy. Intratumoral hemorrhages are common findings in canine brain tumors, including 30 to 40% of gliomas.^5^, ^27^ Although the presence of intratumoral hemorrhage does not differentiate tumor type or grade, increased tumor vascularity and necrosis are reported to be the most important mechanisms of intratumoral hemorrhage and are also classical features of high‐grade gliomas.^20^, ^27^ Many of these tumors will have abnormally dilated, thin‐walled vessels as well.^27^ It is therefore not surprising that intratumoral gradient echo signal voids were related to an increased risk of hemorrhage after biopsy, and serves as an important MRI finding in assessing risk associated with SBB.

When investigating the tumor size, location, biopsy needle path length, and the number of biopsies performed, there were no differences between cases and controls. In addition, we observed no significant influences of imaging features of mass effect on the development of AE. Brain herniation, ventricular distortion, and midline shift are associated with increased intracranial pressure, and as tumor size increases, so does intracranial pressure.^20^, ^21^, ^28^ Historically, there has been concern that SBB could precipitate herniation and exacerbate intracranial hypertension, ultimately leading to AE, although quantitative evidence supporting this is lacking in both people and in veterinary medicine.^12^, ^29^ Similar to previous reports, we also found that pneumocephalus is common finding on imaging studies after SBB, and is frequently asymptomatic.^13^, ^14^ Our findings emphasize the relative safety of SBB in dogs despite tumor size or location.

There are a number of limitations which are largely related to the retrospective nature and design of the study. First, the limited number of patients included in the study could have affected the significance of some of the findings. Second, the majority of tumors in this study were gliomas. Risk factors for AE in gliomas could be different than other tumors. However, in people there does not appear to be a correlation of AE with tumor type.^30^, ^31^ Although the frequency and types of AE seen in our study are similar to previous reports in dogs, our study design selected for those cases that recovered from SBB and had postbiopsy imaging of the brain performed and thus intentionally did not include AE resulting in death.^11^, ^12^, ^13^, ^14^ This and other studies indicate that fatal AE associated with SBB are uncommon, but when they occur are frequently because of catastrophic intracranial hemorrhage that may require transfusion and emergent neurosurgical intervention.^11^, ^12^, ^13^ Finally, imaging assessments for our study were not standardized. MRI field strength is known to cause significant alterations in relaxation and low field strength images can be misleading.^32^ It is also possible that some of the MRI characteristics involving contrast enhancement investigated might be misrepresented, but as relative contrast should not be affected by field strength, this is thought to be improbable.^33^ As we performed CT in immediate proximity to SBB and did not obtain MRI on all dogs in the postbiopsy setting, the frequency with which SBB was associated with intracranial hemorrhage could have been underestimated, or cases with delayed intracranial hemorrhage may not have been identified. However, in humans, CT and MRI are considered equivalent for the diagnosis of acute intracerebral hemorrhage,^34^ and given the clinical outcomes observed in this study, we consider it unlikely that our results are misrepresentative of dogs that develop clinically important intracranial hemorrhage after SBB.

## 5 CONCLUSION

Stereotactic brain biopsy was performed safely in dogs with brain tumors. Overall, 27% (8/29) of dogs recovering from SBB displayed AE characterized by neurological decline, which was mild and resolved within 7 days in 88% (7/8) of cases. Dogs with T2W‐heterogeneous tumors and platelet counts <185 000 mm^3^ are at risk for developing AE associated with SBB. Further investigations are needed to characterize and determine when intracranial hemorrhage on imaging studies performed after SBB is clinically important.

## CONFLICT OF INTEREST DECLARATION

Authors declare no conflict of interest.

## OFF‐LABEL ANTIMICROBIAL DECLARATION

Authors declare no off‐label use of antimicrobials.

## INSTITUTIONAL ANIMAL CARE AND USE COMMITTEE (IACUC) OR OTHER APPROVAL DECLARATION

Authors declare no IACUC or other approval was needed.

## HUMAN ETHICS APPROVAL DECLARATION

Authors declare human ethics approval was not needed for this study.

## Supporting information


**Appendix S1** Supporting Information.
