# Supplementary material for: Risk factors for adverse events occurring after recovery from stereotactic brain biopsy in dogs with primary intracranial neoplasia
Source: J Vet Intern Med. 2020 Sep 14;34(5):2021–8. doi: 10.1111/jvim.15885 (PMC7517515; doi:10.1111/jvim.15885)
Supplement: Supplementary file 1 — Appendix S1 Supporting Information. [file JVIM-34-2021-s001.pdf]

Small Animal Karnofsky Performance Score (KPS)

KPS Score	Description
100	Normal, no complaints, no evidence of disease. Normal ADL; no clinically apparent alterations present (includes asymptomatic tumor).
90	Able to execute normal activity. Normal ADL, clinical signs of disease or physical examination abnormalities subclinical/minor and localized in one organ system. Includes structural epilepsy with normal interictal neurological examination, or subclinical neurological examination abnormalities.
80	Normal activity with effort, some signs of disease obvious. Includes neurological abnormalities that are visible to or reported by owner.
70	Cares for self but unable to perform ADL. At least one organ with major signs of disease. Mildly lethargic over baseline (lethargy grade 1), normal appetite. Includes Frankel Grade 4 SCI.
60	Requires occasional assistance to care for self. At least one organ with major signs of disease. Moderately lethargic over baseline (lethargy grade 2), mildly decreased appetite and/or water intake. <5% BW loss. Includes Frankel Grade 3 SCI.
50	Requires considerable assistance and constant medical care. Lethargy grade 2, moderately decreased appetite, normal water intake. Loss of 5-10% BW. Recumbent, assisted feeding, or Frankel Grades 2, 1, or 0 SCI present.
40	Severely restricted in ADL. Severely decreased appetite. Spontaneous water intake slightly reduced, but without clinical signs of dehydration. Loss of 5-10% BW. Hospitalization recommended, or MGCS <14.
30	Severely disabled, hospitalization indicated but death not imminent. Lethargy grade 3, anorexia or severely diminished water intake with clinical evidence of dehydration. Loss of ≤10% BW or MGCS 10-14.
20	Very sick, hospitalization with active treatment necessary. Severely disabled, all ADL absent, lethargy grade 4, clinically dehydrated, assisted feeding required, or ≥ 3 organs systems with major signs of disease. Loss of > 10% BW or MGCS <10.
10	Moribund/comatose; fatal process progressing rapidly. Patient may require advanced life support measures.
0	Dead

ADL= activities of daily living
BW= body weight
MGCS= modified Glasgow coma score
SCI= spinal cord injury
